# Inverse association of *Helicobacter pylori cag*PAI genotypes with risk of cardia and non‐cardia gastric adenocarcinoma

**DOI:** 10.1002/cam4.2390

**Published:** 2019-07-05

**Authors:** Seyedeh Zahra Bakhti, Saeid Latifi‐Navid, Saber Zahri, Abbas Yazdanbod

**Affiliations:** ^1^ Department of Biology, Faculty of Sciences University of Mohaghegh Ardabili Ardabil Iran; ^2^ Digestive Diseases Research Center Ardabil University of Medical Sciences Ardabil Iran

**Keywords:** *cag*PAI genotypes, CGA, DGA, *Helicobacter pylori*, IGA, *vacA* c2

## Abstract

Iran is a high‐risk country for cardia gastric adenocarcinoma (CGA) in Central Asia, with an incidence rate five times the average global rate, and shows a high infection rate for *Helicobacter pylori* (69%). The aim was to examine the associations of multiple *H. pylori cag*PAI genotypes (ie *cagH*, *cagL*, *cagG*, and *orf17*) with the risk of CGA, non‐CGA, and different histological types of GA in Iran. A large number of *H. pylori* strains (N = 336) were successfully cultured and genotyped. Histopathological evaluations were performed. The analysis showed an inverse association between the *cagH^+^* genotype and the risk of CGA and intestinal‐type gastric adenocarcinoma (IGA) (adjusted ORs; 0.312 and 0.283, respectively), where the controls were nontumors. The *orf17* ^+^ genotype decreased the risk of non‐CGA and diffuse‐type gastric adenocarcinoma (DGA)(adjusted ORs; 0.310 and 0.356, respectively). When the controls were those with nonatrophic gastritis, the *cagG* ^+^ genotype was negatively associated with the risk of CGA, non‐CGA, IGA, and DGA (adjusted ORs; 0.324, 0.366, 0.306, and 0.303, respectively). We did not find such a significant association for the *cagL^+^* genotype in multiple logistic regression analysis. Combination of the *vacA* c2 and *cag*PAI genotypes further decreased the risk estimates for GAs. This study showed the reverse association of *H. pylori cag*PAI genotypes—*cagH* ^+^ and *cagG* ^+^—with the risk of CGA in male patients aged ≥ 55 in Iran. Presence of the *vacA* c2 genotype in combination with *cag*PAI genotypes showed strong inverse associations with the risk of CGA and non‐CGA. These findings may reveal a coordinated relationship between the *vacA* c2 and *cag*PAI genotypes.

## 1 INTRODUCTION

Gastric adenocarcinoma GA) is the fifth common malignancy in the world and is more common in men than in women. GA has been categorized based on the anatomical site of the tumor into two types; cardia gastric adenocarcinoma CGA) and non‐cardia gastric adenocarcinoma NCGA).^1^ In 2012, approximately 951 000 new cases of GA were identified in the world. Of these, 27% and 73% were CGA and NCGA, respectively.^2^ Epidemiological studies have shown that NCGA is strongly associated with *Helicobacter pylori* infection^2^, ^3^ such that 77% of the cases of NCGA are due to infection with this bacterium.^4^ In a meta‐analysis study, *H. pylori* has been suggested as a potential risk factor for increasing the risk of CGA in high‐risk areas. However, it shows a reverse link with the risk of CGA in low‐risk settings.^5^ Iran is a high‐risk country for CGA in Central Asia,^2^ with an incidence rate of five times the average global rate,^2^, ^6^ and shows a high infection rate for *H. pylori* infection 69%).^7^ A considerable heterogeneity among *H. pylori* virulence genes may reflect the differences in the incidence of topographical subtypes and histological characteristics of the tumor.^8^, ^9^

The *cag* pathogenicity island (*cag*PAI) is one of the most important virulence factors in *H. pylori* that encodes a type IV secretion system (T4SS) and has a clear correlation with the progression of adenocarcinoma. The T4SS translocates the virulence factor cytotoxin‐associated gene A CagA) protein into gastric epithelial cells^10^ in which it plays an important role in the onset of GA. The T4SS binding to integrin receptors on gastric epithelial cells is a critical stage for translocation and delivery process of CagA protein. CagL is a pilus protein (26 kDa) and a specialized component for the T4SS that is able to bind to *α*5*β*1 integrin receptor by the Arg‐ Gly‐Asp RGD) motif.^11^ CagH is another integrin‐binding protein of the T4SS with a molecular weight of 39 kDa and consists of 370 amino acids. It has a weak sequence similarity to CagL and is essential for the induction of IL‐8 secretion and CagA translocation into host gastric epithelial cells.^12^ Another gene of *cag*PAI, *cagG*, is located upstream of the *cagA *gene and encodes a protein with 142 amino acids. The *cagG* mutant strains are not capable of delivering CagA into the host cells.^10^, ^13^ The *orf17* gene is another *cag*PAI gene (in *cag*PAI II) that has homology (36% identity) to one of the genes of the *Dickeya zeae* bacterium. Although the *orf17* gene has no meaningful relationship with GC, it increases the risk of peptic ulceration in Iran.^14^

Some studies have shown that strains carrying the *cagA* gene are associated with a high risk of NCGA.^15^, ^16^, ^17^, ^18^, ^19^ and CGA.^20^ A significant association between *vacA* and NCGA, but not CGA, was also found.^20^, ^21^ In another study, both CagA and VacA showed a relationship with an increased risk of intestinal‐type gastric adenocarcinoma IGA) and diffuse‐type gastric adenocarcinoma DGA).^21^ Furthermore, we identified the fifth polymorphic site at the *vacA* gene called c1/‐c2.^22^ The *vacA* c2 vs c1 genotype showed a very strong inverse association with the risk of CGA, non‐CGA, IGA, and DGA in male patients aged ≥ 55 in Iran.^9^ Taken altogether, the etiology of adenocarcinoma of the cardia is not known and still remains controversial. Therefore, the aim was to examine the associations of multiple *H. pylori cag*PAI genotypes (ie *cagH*, *cagL*, *cagG*, and *orf17*) with the risk of CGA and different histological types of GA in Iran.

## 2 MATERIALS AND METHODS

### 2.1 Collection of biopsy specimens

Biopsy samples were collected from 744 patients with digestive diseases referring to endoscopy units in different regions of Iran. Patients were classified into three groups including those with nonatrophic gastritis (NAG), gastric adenocarcinoma (GA), and peptic ulcer (PU) disease. The study was approved by the research Ethics Committee of DDRC. All patients signed written informed consent.

### 2.2 Endoscopy, histological examination, and cultivation

All the gastric biopsies were taken from the antrum and/or the corpus of patients—not from the tumor tissue itself—one biopsy was used to test urease and another to cultivate. For tumor samples, histopathological examination was performed based on the Sydney classification system and according to our previous study.^9^ The anatomical region of the tumor was detected by the endoscopist. The tumors that originated from above the *Z‐*line—the lower one‐third of the esophagus—were considered as esophageal adenocarcinoma, but not CGA, and were thus excluded from all the analyses. The biopsies were cultured on selective culture medium of Brucella agar (Merck, Germany), enriched with 7% defibrinated sheep blood, trimethoprim (5 mg/mL; MP Biomedicals, France), vancomycin (10 mg/mL; Zakaria, Iran), and amphotericin B (4 mg/ mL; Bristol‐Myers Squibb, USA). Plates were incubated under microaerobic conditions containing 5% CO2 and a moisture content above 98% for 4‐10 days at 37°C. The bacterial colonies were identified as *H. pylori* based on negative Gram's staining, showing typical spiral forms, urease, catalase, and oxidase positive tests, and detection of *H. pylori* ‐specific *16S rDNA* using PCR amplification.^9^

### 2.3 DNA extraction and PCR amplification

DNA extraction from *H. pylori* strains was performed using SinaClon DNA extraction kit according to the manufacturer's instructions. Primers listed in Table S1 were used for PCR amplification and sequencing. PCR reaction and cycle parameters were performed as previously described.^14^ PCR products were loaded onto 1% Agarose gel containing safe stain and transferred to a gel Doc to view DNA bands under UV irradiation. For confirmatory purposes, an ABI3700XL DNA sequencer (Applied Biosystems) was used for sequencing the amplified fragments of each gene from 15 strains. The nucleotide sequences were compared with those in GenBank by using the BLAST program (http://www.ncbi.nlm.nih.gov).

### 2.4 Statistical analysis

Simple logistic regression analysis was used to investigate the effect of each factor on the risk of CGA, NCGA, and different histological types of GA. In this analysis, the *Enter* method was used as a model for the input of independent variables. Moreover, multiple logistic regression analysis was performed using the *Forward Stepwise LR* (*Likelihood Ratio*) method with adjustment for sex and a threshold age of ≥55 years. All the two‐sided *P* < 0.05 were considered as significant levels. Data were collected and analyzed using SPSS version 23. To estimate the Q‐value among the tested associations, we used the Q‐value package in *R* version 3.1.1.

## 3 RESULTS

### 3.1 Patients' characteristics and relationship of age and sex with the risk of NCGA and CGA as well as IGA and DGA

A total of 336 *H. pylori* strains were successfully isolated from cultures of the biopsy specimens of Iranian patients (64.6% males and 36.4% females; 48.8% age ≥ 55 years and 50.6% age < 55 years). Based on endoscopic findings, the patients included 127 cases (56 with CGA, 66 with NCGA, and five with both the types of CGA and NCGA) and 209 controls (153 with NAG and 56 with PU) (Table 1). GA was more common in the group of males and the age group of 55 and older. Simple logistic regression analysis showed a significant association between male gender or age ≥ 55 years and the risk of NCGA and CGA as well as IGA and DGA, when the controls were nontumors or those with NAG. (*P* < 0.05; Table S2).

**Table 1:** Characteristics of patients enrolled in this study

Characteristics	No. of patients (%)
Age groups	
≥55	164/336 (48.8)
<55	170/336 (50.6)
No data	2/336 (0.6)
Sex groups	
Males	217/336 (64.6)
Females	119/336 (36.4)
Types of gastroduodenal diseases	
Control	209/336 (62.2)
Nonatrophic gastritis	153/209 (73.2)
Peptic ulcer	56/209 (26.8)
Case	127/336 (37.8)
Cardia gastric adenocarcinoma	56/127 (44.1)
Non‐cardia gastric adenocarcinoma	66/127 (52.0)
Unspecified	5/127 (3.9)
Intestinal‐type adenocarcinoma	75/127 (59.1)
Diffuse‐type adenocarcinoma	39/127 (30.7)
Mucin producing‐type adenocarcinoma	5/127 (3.9)
Signet ring‐type adenocarcinoma	4/127 (3.1)
Adenocarcinoma, poorly differentiated	3/127 (2.4)
Adenocarcinoma, moderate differentiation	1/127 (0.8)
Total	336/336 (100)

### 3.2 Association between the *cag*PAI genotypes *(cagH, cagL, cagG, and orf17)* and NCGA and CGA as well as IGA and DGA

Among 336 strains, the total frequencies of *cagH^+^*, *cagL^+^*, *cagG^+^*, and *orf17^+^* genotypes were 51.2% (172/336), 78.3% (263/336), 62.2% (209/336), and 53.0% (178/336), respectively. In Table 2, the risk estimates for GA in relation to *H. pylori cag*PAI genotypes have been described using simple logistic regression analysis, where the controls were nontumors (patients with either NAG or PU). The simple logistic regression analysis demonstrated that the *cagH^+^*, *cagL^+^*, *cagG^+^*, and *orf17^+^* genotypes had significant reverse associations with risk of both CGA and the NCGA; the OR (95% CI) for *cagH^+^* was 0.260 (0.138‐0.490) and 0.318 (0.179‐0.568), respectively*, cagL^+^* 0.326 (0.166‐0.641) and 0.335 (0.176‐0.637), respectively, *cagG^+^* 0.375 (0.205‐0.367) and 0.294 (0.166‐0.552), respectively, and *orf17^+^* 0.293 (0.158‐0.546) and 0.327 (0.184‐0.580), respectively. Based on the results of the simple logistic regression analysis (Table 3), when the controls were NAG, the *cagH^+^*, *cagL^+^*, *cagG^+^*, and *orf17^+^* genotypes were inversely associated again with both CGA and the NCGA; the OR (95% CI) for *cagH^+^* was 0.313 (0.163‐0.603) and 0.385 (0.211‐0.701), respectively, *cagL^+^* 0.455 (0.230‐0.904) and 0.468 (0.244‐0.898), respectively, *cagG^+^* 0.342 (0.181‐0.647) and 0.269 (0.150‐0.482), respectively, and *orf17^+^* 0.369 (0.195‐0.700) and 0.411 (0.227‐0.745), respectively.

**Table 2:** Risk estimates for CGA, NCGA, IGA, and DGA in relation to *Helicobacter pylori cag*PAI genotypes in a simple logistic regression analysis, where the controls were nontumors

Genotypes	Control^a^ = 209 No.(%)	Cardia gastric adenocarcinoma				Non‐cardia gastric adenocarcinoma				Intestinal‐type adenocarcinoma				Diffuse‐type adenocarcinoma			
		Case = 56 No.(%)	Q‐value^b^	OR^c^	95% CI^d^	Case = 66 No.(%)	Q‐value	OR	95% CI	Case = 75 No.(%)	Q‐value	OR	95% CI	Case = 39 No.(%)	Q‐value	OR	95% CI
*cagH* status																	
*cagH^+^*	131 (62.7)	17 (30.4)	**1.2e‐4** e	**0.260**	**0.138‐0.490**	23 (34.8)	**1.8e‐4**	**0.318**	**0.179‐0.568**	21 (28.0)	**2.6e‐6**	**0.232**	**0.130‐0.412**	15 (38.5)	**1.1e‐2**	**0.372**	**0.184‐0.752**
*cagH^―^*	78 (37.3)	39 (69.6)	1 (ref)	1 (ref)	1 (ref)	43 (65.2)	1 (ref)	1 (ref)	1 (ref)	54 (72.0)	1 (ref)	1 (ref)	1 (ref)	24 (61.5)	1 (ref)	1 (ref)	1 (ref)
*cagL* status																	
*cagL^+^*	179 (85.6)	37 (66.1)	**1.5e‐3**	**0.326**	**0.166 −0.641**	44 (66.7)	**8.3e‐4**	**0.335**	**0.176‐0.637**	50 (66.7)	**5.1e4**	**0.335**	**0.181‐0.621**	27 (69.2)	**1.4e‐2**	**0.377**	**0.172‐0.824**
*cagL^―^*	30 (14.4)	19 (33.9)	1 (ref)	1 (ref)	1 (ref)	22 (33.3)	1 (ref)	1 (ref)	1 (ref)	25 (33.3)	1 (ref)	1 (ref)	1 (ref)	12 (30.8)	1 (ref)	1 (ref)	1 (ref)
*cagG* status																	
*cagG^+^*	152 (72.7)	28 (50.0)	**1.5e‐3**	**0.375**	**0.205‐0.367**	29 (43.9)	**1.1e‐4**	**0.294**	**0.166‐0.552**	33 (44.0)	**2.4e‐5**	**0.295**	**0.170‐0.510**	17 (43.6)	**2.1e‐3**	**0.290**	**0.144‐0.585**
*cagG^―^*	57 (27.3)	28 (50.0)	1 (ref)	1 (ref)	1 (ref)	37 (56.1)	1 (ref)	1 (ref)	1 (ref)	42 (56.0)	1 (ref)	1 (ref)	1 (ref)	22 (56.4)	1 (ref)	1 (ref)	1 (ref)
*orf17* status																	
*orf17^+^*	133 (63.6)	19 (33.9)	**2.1e‐4**	**0.293**	**0.158‐0.546**	24 (36.4)	**1.8e‐4**	**0.327**	**0.184‐0.580**	26 (34.7)	**3.06e‐5**	**0.303**	**0.174‐0.527**	16 (41.0)	**1.2e‐2**	**0.398**	**0.198‐0.799**
*orf17^―^*	76 (36.4)	37 (66.1)	1 (ref)	1 (ref)	1 (ref)	42 (63.6)	1 (ref)	1 (ref)	1 (ref)	49 (65.3)	1 (ref)	1 (ref)	1 (ref)	23 (59.0)	1 (ref)	1 (ref)	1 (ref)

a Non tumors.

b False discovery rate‐adjusted *P*‐value.

c Odds ratio.

d Confidence interval.

e Boldface data indicate statistically significant results.

**Table 3:** Risk estimates for CGA, NCGA, IGA, and DGA in relation to *H. pylori cag*PAI genotypes in a simple logistic regression analysis, where the controls were those with non‐atrophic gastritis

Genotypes	Control^a^^ ^= 153 No.(%)	Cardia gastric adenocarcinoma				Non‐cardia gastric adenocarcinoma				Intestinal‐type adenocarcinoma				Diffuse‐type adenocarcinoma			
		Case = 56 No.(%)	Q‐value^b^	OR^c^	95% CI^d^	Case = 66 No.(%)	Q‐value	OR	95% CI	Case = 75 No.(%)	Q‐value	OR	95% CI	Case = 39 No.(%)	Q‐value	OR	95% CI
*cagH* status																	
*cagH* ^*+*^	89(58.2)	17(30.4)	**1.9e‐3** e	**0.313**	**0.163‐0.603**	23(34.8)	**1.7e‐3**	**0.385**	**0.211‐0.701**	21(28.0)	**5.8e‐5**	**0.280**	**0.154‐0.508**	15(38.5)	5.9e‐2	0.449	0.219‐0.924
*cagH* ^*―*^	64(41.8)	39(69.6)	1(ref)	1(ref)	1(ref)	43(65.2)	1(ref)	1(ref)	1(ref)	54(72.0)	1(ref)	1(ref)	1(ref)	24(61.5)	1(ref)	1(ref)	1(ref)
*cagL* status																	
*cagL* ^*+*^	124(81.0)	37(66.1)	**2.4e‐2**	**0.455**	**0.230‐0.904**	44(66.7)	**2.2e‐2**	**0.468**	**0.244‐0.898**	50(66.7)	**1.7e‐2**	**0.468**	**0.250‐0.876**	27(69.2)	1.1e‐1	0.526	0.239‐1.161
*cagL* ^*―*^	29(19.0)	19(33.9)	1(ref)	1(ref)	1(ref)	22(33.3)	1(ref)	1(ref)	1(ref)	25(33.3)	1(ref)	1(ref)	1(ref)	12(30.08	1(ref)	1(ref)	1(ref)
*cagG* status																	
*cagG* ^*+*^	114(74.5)	28(50.0)	**1.9e‐3**	**0.342**	**0.181‐0.647**	29(43.9)	**2.1e‐5**	**0.268**	**0.146‐0.492**	33(44.0)	**4.0e‐5**	**0.269**	**0.150‐0.482**	17(43.6)	**1.4e‐3**	**0.264**	**0.127‐0.549**
*cagG* ^*―*^	39(25.5)	28(50.0)	1(ref)	1(ref)	1(ref)	37(56.1)	1(ref)	1(ref)	1(ref)	42(56.0)	1(ref)	1(ref)	1(ref)	22(56.4)	1(ref)	1(ref)	1(ref)
*orf17* status																	
*orf17* ^*+*^	89(58.2)	19(33.9)	**3.02e‐3**	**0.369**	**0.195‐0.700**	24(36.4)	**3.4e‐3**	**0.411**	**0.227‐0. 745**	26(34.7)	**1.3e‐3**	**0.382**	**0.215‐0.677**	16(41.0)	7.6e‐2	0.500	0.245‐1.022
*orf17* ^*―*^	64(41.8)	37(66.1)	1(ref)	1(ref)	1(ref)	42(63.6)	1(ref)	1(ref)	1(ref)	49(65.3)	1(ref)	1(ref)	1(ref)	23(59.0)	1(ref)	1(ref)	1(ref)

^a^Non‐atrophic gastritis; ^b^False discovery rate‐adjusted *P*‐value; ^c^Odds ratio; ^d^Confidence interval; ^e^Boldface data indicate statistically significant results.

When further analysis was performed based on the histological characteristics of the tumor, the frequency of the *cagH* ^+^, *cagL* ^+^, *cagG* ^+^, and *orf17* ^+^ genotypes in patients with IGA (28.0%, 66.7%, 44.0%, and 34.7%, respectively) and DGA (38.5%, 69.2%, 43.6%, and 41.0%, respectively) was lower than in those with no tumors (62.7%, 85.6%, 72.7%, and 63.6%, respectively) (Table 2). These genotypes showed significant reverse associations with the risk of IGA (the OR (95% CI) was 0.232 (0.130‐0.412), 0.335 (0.181‐0.621), 0.295 (0.170‐0.510), and 0.303 (0.174‐0.527), respectively) and DGA (the OR (95% CI) was 0.372 (0.184‐0.752), 0.377 (0.172‐0.824), 0.290 (0.144‐0.585), and 0.398 (0.198‐0.799), respectively) in simple logistic regression analysis (Table 3).

Where the controls were those with NAG, the *cagH^+^*, *cagL^+^*, *cagG^+^*, and *orf17^+^* genotypes were significantly associated with a decreased risk of IGA; the OR (95% CI) was 0.280 (0.154‐0.508), 0.468 (0.250‐0.876), 0.269 (0.150‐0.482) and 0.382 (0.215‐0.677), respectively. The *cagG^+^* genotype was inversely associated with the risk of DGA; the OR (95% CI) was 0.264 (0.127‐0.549) (Table 3).

Eventually, in a multiple logistic regression analysis, the *cagH^+^* genotype was negatively correlated with the age‐ and sex‐adjusted risk for CGA and IGA, and the *orf17^+^* genotype for NCGA and DGA, where the controls were nontumors; the OR (95% CI) for *cagH^+^* was 0.312 (0.150‐0.651) and 0.283 (0.148‐0.543), respectively and for *orf17^+^* 0.310 (0.158‐0.607) and 0.356 (0.154‐0.826), respectively. The multiple logistic regression analysis also showed an inverse association between the *cagG^+^* genotype and the adjusted risk for CGA, NCGA, IGA, and DGA, where the controls were those with NAG (the ORs [95% CI] were 0.324 [0.139‐0.759], 0.366 [0.175‐0.762], 0.306 [0.150‐0.625], and 0.303 [0.116‐0.790], respectively) (Table 4).

**Table 4:** Age‐ and sex‐adjusted risk for CGA, NCGA, IGA, and DGA in relation to *Helicobacter pylori cag*PAI genotypes in a multiple logistic regression analysis

Genotypes	Cardia gastric adenocarcinoma			Non‐cardia gastric adenocarcinoma			Intestinal‐type adenocarcinoma			Diffuse‐type adenocarcinoma		
	*P*‐value	OR^a^	95% CI^b^	*P*‐value	OR	95% CI	*P*‐value	OR	95% CI	*P*‐value	OR	95% CI
Gastric adenocarcinoma vs nontumors												
*cagH^+^* vs *cagH^―^*	0.002	0.312	0.150‐0.651	—	—	—	0.000	0.283	0.148‐0.543	—	—	—
*orf17^+^* vs *orf17^―^*	—	—	—	0.001	0.310	0.158‐0.607	—	—	—	0.016	0.356	0.154‐0.826
Gastric adenocarcinoma vs nonatrophic gastritis												
*cagG^+^* vs *cagG^―^*	0.009	0.324	0.139‐0.759	0.007	0.366	0.175‐0.762	0.001	0.306	0.150‐0.625	0.015	0.303	0.116‐0.790

a Odds ratio.

b Confidence interval.

### 3.3 Association between the presence of the* vacA* c2 genotype in combination with the *cag*PAI genotypes *(cagH, cagL, cagG, and orf17)* and NCGA and CGA as well as IGA and DGA

As illustrated in Table 5, when the controls were nontumors, the presence of *vacA* c2 in combination with *cag*PAI genotypes further reduced the risk of both CGA and the NCGA. The OR for *cagH/vacAc2* was 0.109 (0.037‐0.321) and 0.134 (0.054‐0.333), respectively, *cagL/vacAc2* 0.172 (0.078‐0.384) and 0.112 (0.051‐0.250), respectively, *cagG/vacAc2* 0.259 (0.115‐0.582) and 0.119 (0.048‐0.296), respectively, and *orf17/vacAc2* 0.125 (0.046‐0.337) and 0.119 (0.048‐0.296), respectively. Further analysis revealed that there was an inverse relationship between the four combinations of *cag*PAI genotypes with *vacA* c2 genotype and the risk of both IGA and DGA (Table 5). The results of simple logistic regression analysis for the associations of combination genotypes (*vacA* c2 region genotype in combination with the *cag*PAI genotypes) with GA risk are shown in Table 6, where the controls are NAG.

**Table 5:** Risk estimates for CGA, NCGA, IGA, and DGA in relation to *Helicobacter pylori* combination genotypes in a simple logistic regression analysis, where the controls were nontumors

Genotypes	Control^a^ = 174 No.(%)	Cardia gastric adenocarcinoma				Non‐cardia gastric adenocarcinoma				Intestinal‐type adenocarcinoma				Diffuse‐type adenocarcinoma			
		Case = 36 No.(%)	Q‐value^b^	OR^c^	95% CI^d^	Case = 45 No.(%)	*Q*‐ value	OR	95% CI	Case = 52 No.(%)	*Q*‐ value	OR	95% CI	Case = 25 No.(%)	*Q*‐ value	OR	95% CI
*cagH/vacAc2*	93(53.4)	4(11.1)	**7.7e‐5** e	**0.109**	**0.037‐0.321**	6(13.3)	**1.5e‐5**	**0.134**	**0.054‐0.333**	6(11.5)	**2.0e‐6**	**0.114**	**0.046‐0.280**	4(16.0)	**1.5e‐3**	**0.166**	**0.055‐0.503**
*cagL/vacAc2*	120(69.0)	10 (27.8)	**6.4e‐5**	**0.172**	**0.078‐0.384**	9(20.0)	**3.2e‐7**	**0.112**	**0.051‐0.250**	13(25.0)	**5.3e‐7**	**0.150**	**0.074‐0.304**	4(16.0)	**6.4e‐5**	**0.086**	**0.028‐0.262**
*cagG/vacAc2*	98(56.3)	9(25.0)	**1.08e‐3**	**0.259**	**0.115‐0.582**	6(13.3)	**6.6e‐6**	**0.119**	**0.048‐0.296**	9(17.3)	**5.0e‐6**	**0.162**	**0.075‐0.354**	4(16.0)	**9.8e‐4**	**0.148**	**0.049‐0.448**
*orf17/vacAc2*	98(56.3)	5(13.9)	**7.7e‐5**	**0.125**	**0.046‐0.337**	6(13.3)	**6.6e‐6**	**0.119**	**0.048‐0.296**	6(11.5)	**1.2e‐6**	**0.101**	**0.041‐0.249**	4(16.0)	**9.8e‐4**	**0.148**	**0.049‐0.448**

a Non tumors.

b False discovery rate‐adjusted *P*‐value.

c Odds ratio.

d Confidence interval

e Boldface data indicate statistically significant results.

**Table 6:** Risk estimates for CGA, NCGA, IGA, and DGA in relation to *Helicobacter pylori* combination genotypes in a simple logistic regression analysis, where the controls were those with nonatrophic gastritis

Genotypes	Control^a^ = 120 No.(%)	Cardia gastric adenocarcinoma				Non‐cardia gastric adenocarcinoma				Intestinal‐type adenocarcinoma				Diffuse‐type adenocarcinoma			
		Case = 36 No.(%)	Q‐value^b^	OR^c^	95% CI^d^	Case = 45 No.(%)	*Q*‐ value	OR	95% CI	Case = 52 No.(%)	*Q*‐ value	OR	95% CI	Case = 25 No.(%)	*Q*‐ value	OR	95% CI
*cagH/vacAc2*	61(50.8)	4(11.1)	**2.2e‐4** e	**0.121**	**0.040‐0.363**	6(13.3)	**6.1e‐5**	**0.149**	**0.059‐0.378**	6(11.5)	**1.1e‐5**	**0.126**	**0.050‐0.318**	4(16.0)	**3.2e‐3**	**0.184**	**0.060‐0.569**
*cagL/vacAc2*	79(65.8)	10 (27.8)	**2.2e‐4**	**0.200**	**0.0.088‐0.454**	9(20.0)	**4.0e‐6**	**0.130**	**0.057‐0.295**	13(25.0)	**5.3e‐6**	**0.173**	**0.083‐0.360**	4(16.0)	**2.5e‐4**	**0.099**	**0.032‐0.307**
*cagG/vacAc2*	70(58.3)	9(25.0)	**7.8e‐4**	**0.238**	**0.103‐0.550**	6(13.3)	**8.0e‐6**	**0.110**	**0.043‐0.279**	9(17.3)	**5.3e‐6**	**0.150**	**0.067‐0.334**	4(16.0)	**1.0e‐3**	**0.136**	**0.044‐0.421**
*orf17/vacAc2*	64(53.3)	5(13.9)	**2.2e‐4**	**0.141**	**0.051‐0.388**	6(13.3)	**3.2e‐5**	**0.135**	**0.053‐0.342**	6(11.5)	**5.3e‐6**	**0.114**	**0.045‐0.287**	4(16.0)	**2.4e‐3**	**0.167**	**0.054‐0.515**

a Nonatrophic gastritis.

b False discovery rate‐adjusted *P*‐value.

c Odds ratio.

d Confidence interval.

e Boldface data indicate statistically significant results.

## 4 DISCUSSION

For men in Iran, the incidence rate of CGA is twice the rate of NCGA, unlike what is observed worldwide.^2^ In the present study, more than 80% of patients in CGA group and more than 70% of patients in NCGA group were males and had age ≥ 55 years. CGA was approximately seven times more common among men (87.5%) than women (12.5%). Furthermore, we found significant associations between male gender or age ≥ 55 and the risk of CGA and NCGA, where the controls were nontumors (ORs = 5.83 vs 2.83 and ORs = 15.51 vs 12.69, respectively) or those with NAG (ORs = 8.75 vs 4.25 and ORs = 17.38 vs 14.224, respectively). Statistical analysis revealed a very strong correlation between age ≥ 55 and DGA compared to IGA. The OR was 47.84 (vs 9.37) when the controls were nontumors. The OR estimate also was 53.603 (vs 10.50) when the controls were those with NAG.

The reason for a higher incidence of CGA in Iran is unknown; however, a high infection rate for *H. pylori* 69%)^7^ and a considerable heterogeneity among *H. pylori* virulence genes may contribute to a high rate of CGA in the Iranian population. In a case‐control study in Northeastern Iran, CagA seropositivity was related to an increased risk of both CGA OR = 1.9) and NCGA OR = 3.4). A significant association was also found between seropositivity to VacA antigen and risk of NCGA OR = 2.8), but not CGA.^20^ However, several studies from different parts of the world have shown that there was no association between CagA antibodies and CGA.^21^, ^23^ There was no significant statistical correlation between the *cagA^+^* genotype and the risk of CGA.^9^, ^21^ However, other studies revealed an inverse association between *cagA^+^* strains and the development of CGA.^24^, ^25^

The *cagH^+^* genotype showed significant reverse associations with the risk of both CGA and the NCGA. Also when the controls were NAG, the *cagH ^+^* genotype was inversely associated again with both CGA.

It has been demonstrated that more than 85% of the *H. pylori* strains isolated from patients in India, Malaysia, Taiwan, Iran, and Singapore were *cagL* positive.^14^, ^26^, ^27^ No association was found between this genotype and clinical outcomes.^27^, ^28^, ^29^, ^30^, ^31^ These results were consistent with the results of the Raei et al study for GC, but not for PU OR = 10.950).^14^ In the present study, 78.3% of strains had the *cagL^+^* genotype and the results of logistic regression analysis showed a significant reverse association between this genotype and the risk of CGA and NCGA, whether the controls were nontumors or those with NAG.

Various studies from Asian countries such as China, Korea, Japan, and Iran showed that although there was a high prevalence of *cagG^+^* genotype in these populations (91.7%, 86.7%, 97%, and 71.5%, respectively), no significant association with gastrointestinal diseases was found.^14^, ^32^, ^33^ In the present study, the *cagG^+^* genotype was the most frequent after the *cagL^+^* genotype and showed a significant, but inverse association with the risk of both CGA and NCGA, whether the controls were nontumors or those with NAG.

The *orf17* genotype, like the *cagH* genotype, has not been well studied at the genomic level; only one study from Iran revealed that the *orf17* genotype had a remarkable relationship with an increased risk of PU (OR = 2.504) but not GC.^14^ However, in the present study, a remarkable but negative relationship was found between the *orf17^+^* genotype and the risk of CGA and NCGA, whether the controls were nontumors or those with NAG.

The associations of some *H. pylori* genotypes and their role in the development of histological types of GC have been confirmed in previous studies.^21^, ^34^ In a study from Sweden, CagA and VacA antibodies were linked to a heightened risk of both intestinal‐ (ORs = 6.0 and 3.7, respectively) and diffuse‐ (ORs = 20.6 and 3.9, respectively) type GC.^21^ In the present study, significant reverse associations of the *cagH^+^*, *cagL^+^*, *cagG^+^*, and *orf17^+^* genotypes with the risk of IGA and DGA were found, where the controls were nontumors. The results of simple logistic regression analysis, where the controls were those with NAG, demonstrated that the *cagH^+^*, *cagL^+^*, *cagG^+^*, and *orf17^+^* genotypes were associated with a decreased risk of IGA, and only *cagG^+^* genotype had a significant reverse relationship with the risk of DGA.

Eventually, in the multiple logistic regression analysis, after being adjusted for confounding factors, the *cagG^+^* genotype was associated with a reduced risk of CGA and NCGA as well as IGA and DGA (the adjusted ORs = 0.324, 0.366, 0.306, and 0.303, respectively), where the controls were those with NAG. However, when the control groups were nontumors, there were variations between the associations of *cag*PAI genotypes with the risk of CGA or non‐CGA and IGA or DGA, so that the the *cagH^+^* genotype had a strong correlation with a reduced risk of CGA and IGA (ORs = 0.312 and 0.283, respectively), and the *orf17^+^* genotype had a negative correlation with the risk of NCGA and DGA (ORs = 0.310 and 0.356, respectively).

These findings are the first report on a reverse association of *H. pylori cag*PAI genotypes with the risk of CGA in male patients aged ≥ 55 in Iran. In the present study, the presence of the *vacA* c2 genotype in combination with *cag*PAI genotypes showed strong inverse associations with the risk of CGA. These findings may reveal a coordinated relationship between the *vacA* c2 and *cag*PAI genotypes; however, it is still vague and requires more research.

## CONFLICT OF INTEREST

No conflict of interest to be declared.

## AUTHORS’ CONTRIBUTIONS

SL‐N, SZ and AY conceived and designed the experiments; SZB and AY performed the experiments; SL‐N and SZB analyzed the data; SZB and SL‐N wrote the manuscript. All authors read and approved the final manuscript.
