# Supplementary material for: Inverse association of Helicobacter pylori cagPAI genotypes with risk of cardia and non‐cardia gastric adenocarcinoma
Source: Cancer Med. 2019 Jul 5;8(10):4928–37. doi: 10.1002/cam4.2390 (PMC6712521; doi:10.1002/cam4.2390)
Supplement: Supplementary file 1 [file CAM4-8-4928-s001.doc]

*Inverse Association of Helicobacter pylori cagPAI Genotypes with Risk of Cardia and Non-Cardia Gastric Adenocarcinoma*

**Seyedeh Zahra Bakhti^1^, Saeid Latifi-Navid^1, *^, Saber Zahri^1^, Abbas Yazdanbod^2^**

*^1^Department of Biology, Faculty of Sciences, University of Mohaghegh Ardabili, Ardabil, 56199-11367 Iran*

*^2^ Digestive Diseases Research Center, Ardabil University of Medical Sciences, Ardabil, Iran*

***Correspondence to:** Saeid Latifi-Navid, Ph.D., Department of Biology, Faculty of Sciences, University of Mohaghegh Ardabili, Ardabil, 56199-11367 Iran, Telefax: +98-45-33514701, E-mail: [*s_latifi@uma.ac.ir*](mailto:s_latifi@uma.ac.ir)

**Supplementary Table S1. The sequence and characteristics of primers used for PCR**

| **Genes** | **Primers** | **Sequences (5ʹ→3ʹ)** | **Size of PCR products (bp)** | | **Optimized annealing temperature (°C)** |
| --- | --- | --- | --- | --- | --- |
| ***16 S rDNA*** | HP1 | GCAATCAGCGTCAGTAATGTTC | | 519 | 56 |
|  | HP2 | GCTAAGAGATCAGCCTATGTCC | |  |  |
| ***cagH*** |  |  | |  |  |
|  | Forward | ATGGCAGGTACACAAGCTAT | | 1113 | 52 |
|  | Reverse | TCACTTCACGATTATTTTAG | |  |  |
| ***cagL*** |  |  | |  |  |
|  | Forward  Reverse | AAAACACTCGTGAAAAATACCATATC TCGCTTCAAAATTGGCTTTC | | 263 | 54 |
| ***cagG*** |  |  | |  |  |
|  | Forward | TTATAAAATTAAATTACTATTTGC | | 398 | 50 |
|  | Reverse | GTGGTAAAAACGATGAATCTG | | 593 | 51 |
| ***orf17*** |  |  | |  |  |
|  | Forward | CTTGATTGATGAAAATTTGGTTG | | 546 | 50 |
|  | Reverse | TTAGTGATATATTCATAATTTTCC | |  |  |

| **Supplementary Table S2.** Association between age and sex and the anatomic origin and histological type of the tumors | | | | | | | | | | | | | | | | | | | | | |
| --- | --- | --- | --- | --- | --- | --- | --- | --- | --- | --- | --- | --- | --- | --- | --- | --- | --- | --- | --- | --- | --- |
| **Genotypes** |  | **Cardia gastric adenocarcinoma** | | | | | **Non-cardia gastric adenocarcinoma** | | | | | **Intestinal type adenocarcinoma** | | | | | **Diffuse type adenocarcinoma** | | | | |
|  | **Control**  **No.(%)** | **Case**  **No.(%)** | ***P* value** | **OR^a^** | **95% CI^b^** | **Total**  **No.(%)** | **Case**  **No.(%)** | ***P* value** | **OR** | **95% CI** | **Total No.(%)** | **Case**  **No.(%)** | ***P* value** | **OR** | **95% CI** | **Total No.(%)** | **Case**  **No.(%)** | ***P* value** | **OR** | **95% CI** | **Total No.(%)** |
| **Gastric adenocarcinomas**  **vs. non-tumors** | | | |  |  |  |  |  |  |  |  |  |  |  |  |  |  |  |  |  |  |
| **Sex** |  |  |  |  |  |  |  |  |  |  |  |  |  |  |  |  |  |  |  |  |  |
| **Male** | 114(54.5) | 49(87.5) | **0.00^c^** | **5.83** | **2.52-13.47** | 163(61.5 | 51(77.3) | **0.001** | **2.83** | **1.49-5.35** | 165(60.0) | 60(80.0) | **.000** | **3.33** | **1.77-6.24** | 174(61.3) | 32(82.1) | **0.002** | **3.81** | **1.60-9.02** | 146(58.9) |
| **Female** | 95(45.5) | 7(12.5) | 1(ref) | 1(ref) | 1(ref) | 102(38.5) | 15(22.7) | 1(ref) | 1(ref) | 1(ref) | 110(40.0) | 15(20.0) | 1(ref) | 1(ref) | 1(ref) | 110(38.7) | 7(17.0) | 1(ref) | 1(ref) | 1(ref) | 102(41.1) |
| **Age, y** |  |  |  |  |  |  |  |  |  |  |  |  |  |  |  |  |  |  |  |  |  |
| **≥55** | 58(27.9) | 48(85.7) | **0.00** | **15.51** | **6.92-34. 79** | 106(40.2) | 54(83.1) | **0.00** | **12.69** | **6.20-25.97** | 112(41.0) | 58(78.4) | **0.000** | **9.37** | **4.98-17.62** | 116(41.1) | 37(94.9) | **0.00** | **47.84** | **11.16-204. 94** | 95(38.5) |
| **<55** | 150(72.1) | 8 (14.3) | 1(ref) | 1(ref) | 1(ref) | 158(59.8) | 11(16.9) | 1(ref) | 1(ref) | 1(ref) | 161(59.0) | 16(21.6) | 1(ref) | 1(ref) | 1(ref) | 166(58.9) | 2(5.1) | 1(ref) | 1(ref) | 1(ref) | 152(61.5) |
| **Gastric adenocarcinomas**  **vs. non-atrophic gastritis** | | | |  |  |  |  |  |  |  |  |  |  |  |  |  |  |  |  |  |  |
| **Sex** |  |  |  |  |  |  |  |  |  |  |  |  |  |  |  |  |  |  |  |  |  |
| **Male** | 68(44.4) | 49(87.5) | **0.00** | **8.75** | **3.72-20.54** | 117(56.0) | 51(77.3) | **0.000** | **4.25** | **2.20-8.20** | 119(54.3) | 60(80.0) | **0.000** | **5.00** | **2.61-9.57** | 128(56.1) | 32(82.1) | **0.000** | **5.714** | **2.37-13.74** | 100(52.1) |
| **Female** | 85(55.6) | 7(12.5) | 1(ref) | 1(ref) | 1(ref) | 92(44.0) | 15(22.7) | 1(ref) | 1(ref) | 1(ref) | 100(45.7) | 15(20.0) | 1(ref) | 1(ref) | 1(ref) | 100(43.9) | 7(17.9) | 1(ref) | 1(ref) | 1(ref) | 92(47.9) |
| **Age, y** |  |  |  |  |  |  |  |  |  |  |  |  |  |  |  |  |  |  |  |  |  |
| **≥55** | 39(26.7) | 48(85.7) | **0.00** | **17.38** | **7.56-39.96** | 87(41.8) | 54(83.1) | **0.000** | **14.224** | **6.76-29.91** | 93(42.9) | 58(78.4) | **0.000** | **10.50** | **5.41-20.37** | 97(42.9) | 37(94.9) | **0.000** | **53.603** | **12.34-232.82** | 76(39.8) |
| **<55** | 113(74.3) | 8(14.3) | 1(ref) | 1(ref) | 1(ref) | 121(58.2) | 11(16.9) | 1(ref) | 1(ref) | 1(ref) | 124(57.1) | 16(21.6) | 1(ref) | 1(ref) | 1(ref) | 129(57.1) | 2(5.1) | 1(ref) | 1(ref) | 1(ref) | 115(60.2) |
| **a: Odds ratio; b: Confidence interval; c: Boldface data indicate statistically significant results.** | | | | | | | | | | | | | | | | | | | | | |
